# Possible adverse events in children treated by manual therapy: a review

**DOI:** 10.1186/1746-1340-18-12

**Published:** 2010-06-02

**Authors:** B Kim Humphreys

**Affiliations:** 1 Professor Chiropractic Medicine, University of Zürich and University Orthopedic Hospital Balgrist. Forchstrasse 340, 8008 Zürich, Switzerland

## Abstract

**Background:**

Pediatric manual therapy is controversial within the medical community particularly with respect to adverse events. Pediatric manual therapy (Ped MT) is commonly used by a number of professions such as chiropractors, osteopaths and naturopaths for a variety of treatments in children. Ped MT interventions range from advice, light touch, massage, through to mobilisation and high velocity spinal manipulation. However, current evidence related to adverse events associated with Ped MT is not well understood.

**Objective:**

To update the clinical research literature from the 2007 report by Vohra, Johnston, Cramer and Humphreys on possible adverse events in children treated by spinal manipulation.

**Methods:**

A review of the clinical research literature from June 2004 until January 2010 as reported in MEDLINE, PubMed and PubMed Central for adverse events specifically related to the treatment of pediatric cases by manual therapy.

**Results:**

Only three new clinical studies, one systematic review with meta-analysis and one evidence report were identified. Two clinical studies reported on chiropractic care and one on osteopathic spinal manipulation in children. The systematic review investigated all studies of adverse events and manual therapy and was not specific for pediatric patients. The evidence review focused on effectiveness of spinal manipulation in a variety of musculoskeletal conditions. No serious or catastrophic adverse events were reported in the clinical studies or systematic review. However for adults, it has been estimated that between 0.003% and 0.13% of manual therapy treatments may result in a serious adverse event. Although mild to moderate adverse events are common in adults, an accurate estimate from high quality pediatric studies is currently not available.

**Conclusions:**

There is currently insufficient research evidence related to adverse events and manual therapy. However, clinical studies and systematic reviews from adult patients undergoing manual therapy, particularly spinal manipulation report that mild to moderate adverse events are common and self limiting. However serious adverse events are rare and much less than for medication commonly prescribed for these problems. More high quality research specifically addressing adverse events and pediatric manual therapy is needed.

## Introduction

The treatment of children with manual therapy (MT) such as spinal manipulation is controversial within the medical community particularly with respect to adverse events [1]. Chiropractors, osteopaths, naturopaths, physical therapists and medical practitioners are the most common health care professionals that may utilize manual therapy for pediatric patients. Of these, chiropractic is the largest complementary and alternative medical (CAM) profession visited by children and the most likely to use manual therapy, especially spinal manipulation [2,3]. As an example, it has been estimated that 30 million children made visits to chiropractors in the United States alone during 1997 [4].

Chiropractic, osteopathic and naturopathic care for children may employ manual therapy for a variety of disorders and conditions ranging from asthma, infantile colic, otitis media, enuresis, birth trauma, to less controversial mechanical back, neck pain and headache [5-7]. The use of pediatric manual therapy (Ped MT) for these health conditions is currently based on low levels of scientific evidence [5-7]. However, a recent comprehensive systematic review of chiropractic care rather than Ped MT only for non-musculoskeletal conditions is more encouraging [8].

In Switzerland where chiropractic in now part of the medical profession, 22% of chiropractors receive weekly to monthly referral of pediatric patients from pediatricians. A 2009 Job Analysis of Swiss chiropractors revealed 91% of those surveyed reported treating patients aged between 6-17 years with 78% treating patients less than 5 years of age (Humphreys BK, Peterson CK, Mühlemann D, Heuter P. Unpublished data).

Nevertheless there are opponents of the use of Ped MT for any pediatric condition citing that it may be harmful or ineffective [9]. Others suggest the controversy is part of an organized conspiracy against CAM professions by the pharmaceutical industry [10]. Regardless, it is important that the research evidence regarding possible adverse events for pediatric patients receiving Ped MT, particularly spinal manipulation, is continually reviewed.

For the purpose of this report, the age range for pediatrics is typically 18 years of age and younger. After Carnes et al (2009, 2010), manual therapy may be described as 'any technique administered manually, using touch, by a trained practitioner for therapeutic purposes.' [11,12]. In the pediatric population, Ped MT may range from advice, light touch, soft tissue massage, passive or active mobilization through to high velocity, low or short amplitude thrust (spinal manipulation) delivered to a spinal joint [5,6,11-13]. Alcantara et al [14] suggest that pediatric spinal manipulation is considerably different compared to adult SMT. Pediatric SMT is typically adapted to the unique biomechanical features of the pediatric spine. The forces delivered are much less than those for an adult patient and may be as little as touch or pressure in infants to a modified thrust in adolescents. Contact points, patient and therapist position, number of areas treated and treatment schedule may all be adapted to suit the development and needs of the pediatric patient [8,14-16].

An adverse event is an untoward or negative side effect resulting from treatment. An adverse event may be categorized as serious (requiring hospitalization, permanent disability or mortality), moderate (transient disability, medical care sought or needed but not hospitalization) and minor (self limited and did not require additional medical care) [15].

The purpose of this review article is to look at the best available evidence related to possible adverse events in children treated by Ped MT.

## Review

### Systematic Review of Adverse Events Associated with Pediatric Spinal Manipulation

The publication by Vohra, Johnston, Cramer and Humphreys [15] remains the most current, comprehensive systematic review of the literature on adverse events associated with pediatric spinal manipulation. Vohra et al [15] performed a comprehensive search of eight major electronic databases including MEDLINE, PubMed, Embase, AMED and MANTIS from inception to 2004. All languages were included. In particular, MEDLINE and PubMed searches covered a period of 58 years from 1966 to 2004. In order for the reports or studies to be included, they must have been primary investigations of spinal manipulation; the study population included participants 18 years of age or younger and data on adverse events was reported. Reports were not limited to any particular healthcare profession.

Adverse events (AE) were categorized as severe, moderate or minor as described previously. Interestingly and controversially, adverse events rated as severe or moderate as the result of a delay in diagnosis and not directly related to the application of spinal manipulation, were also included in the data.

Thirteen reports of adverse events associated with pediatric spinal manipulation were identified over the 58 year period to June 2004. Of these, two were clinical trials [17,18]; four were case studies [19-22]; and seven were case reports [23-29]. Ten reports were in English, two in French and one in German.

Of the 13 reports, nine were categorized as serious adverse events (SAE), one as **a **moderate adverse event (MoAE) and three as minor adverse events (MiAE). Interestingly, of the nine SAE, eight were single case reports and one was a case series involving three children. Therefore all case reports and one of the two case series identified by Vohra et al, [15] involved SAE. It is noteworthy that neither of the two clinical trials [17,18] resulted in any SAE.

### Limitations of Vohra et al Systematic Review [15]

No studies, including systematic reviews, are free of limitations or indeed errors which may affect their results and subsequent contribution to the scientific literature. Nevertheless it is clear that regardless of the limitations, this systematic review is important and provides useful information related to adverse events and pediatric spinal manipulation. The following is a discussion of the major limitations related to the Vohra et al [15] systematic review that is relevant to this current paper.

### Serious Adverse Events (SAE)

The results of the Vohra et al [15] comprehensive literature search covering a 58 year period, involving eight major databases and including all language reports, resulted in nine serious adverse events (SAE) related to Ped MT. However, on further analysis, of the nine SAE, one involved an examination of a patient's traumatized cervical spine by a medical practitioner [29]. Examination of the cervical spine is not done with therapeutic intent thereby excluding it by definition from Ped MT [11,12]. Consequently a total of eight SAE instead of nine SAE should have been reported.

### Misdiagnosis and Delayed Diagnosis

Of the SAE reported, at least three and possibly up to five of these cases had underlying pathologies or conditions (spinal cord astrocytoma, osteogenesis imperfecta, congenital occipitalization) which are clearly contraindications for Ped MT [25,28,29]. SAE understandably resulted from inappropriate application of spinal manipulation in these cases due to a misdiagnosis. The clinical point here is that the association of an adverse event to pediatric SMT arises from a misdiagnosis.

Equally, a delayed diagnosis of a serious underlying pathology or condition needing referral to the appropriate medical practitioner may also result in a SAE or MoAE. A study of 90 chiropractors in the Boston metropolitan area in the United States identified that 17% would continue to be the sole treatment provided in a hypothetical neonate case with a two week history of fever [4]. Clearly this conflicts with current medical guidelines for treatment of pediatric cases with fever and likely will result in a delayed diagnosis and possibly some type of adverse event. As above, the clinical point is that a delayed diagnosis leads to the inappropriate application of SMT and the AE.

Consequently the author suggests that it would be more informative for clinicians and researchers if in addition to the current classification system, delayed diagnosis would be identified if appropriate. Consequently Ped MT given to a patient with a serious underlying pathology leading to a SAE would be classified as SAE (misdiagnosis) rather than simply SAE. Similarly if Ped MT were performed in a case of misdiagnosis which led to a moderate AE, it would be classified as MoAE (misdiagnosis) and so forth. An SAE alone is the result of the treatment without serious underlying pathology. Currently adverse events are lumped together and only by sorting through the details can one arrive at a better understanding of the AE. This is unhelpful to clinicians as it hides the underlying cause of the SAE.

### Update of Research Literature on Possible Adverse Events and Ped MT

One of the common criticisms lodged against the paucity of adverse events and Ped MT in the literature is that the spontaneous reporting of AE leads to underreporting [15,28].

Since the publication of the Vohra et al [15] systematic review, a search of the literature (MEDLINE, PubMed, PubMed Central) from July 2004 until present (January 2010) retrieved three reports related to Ped MT and adverse events. All three studies made an attempt at providing information on prevalence or rates of adverse events in pediatric patients undergoing manual therapy although with variable quality.

Hayes and Bezilla [30] conducted a retrospective review of adverse events (aggravation and complications) in the medical records for pediatric patients receiving Osteopathic SMT (OPSMT). A total of 346 out of 502 files reviewed met their inclusion criteria. Although no serious complications were found associated with OPSMT, 9% (31 patients out of 346) had file entries of clinical aggravation after OPSMT. These could be categorized as minor adverse events such as worsening symptoms, irritability, soreness, headache, behavior problems and pain. The authors' reported that their findings support OPSMT as a safe treatment for the pediatric population [30]. However, a retrospective file review may suffer from numerous methodological flaws and the results should be viewed with caution.

Miller and Benfield [16] published a three year retrospective case file review of all patients younger than three years of age attending the outpatient clinic of the Anglo-European College of Chiropractic in the UK. A total of 781 pediatric cases were retrieved. Of these 699 (89.5%) pediatric cases representing 5242 treatment visits were included with over 77% having received Ped MT. The other 82 (10.5%) were referred for other care. Most of the pediatric patients (n = 574; 73.5%) were 12 weeks of age or younger. The most common age group was between five and eight weeks with the most common presenting complaint being attributed to spinal biomechanical dysfunction manifesting as 'irritability or colic' often attributed to birth trauma. Miller and Benfield [16] reported that over 85% of parents reported improvement in their child's complaints with treatment while just over 14% reported no change.

For adverse events, negative side effects as identified by the parent were reported. A total of seven adverse events out of 697 pediatric patients (two were lost to follow-up) were elicited. All seven involved minor adverse events (crying or increased crying or restlessness or sleeping disturbance) which were transient and did not require medical care.

Miller and Benfield [16] estimate 1% of pediatric patients (one in 749 treatments) suffered from a minor and self limited AE. A detailed description of the AE allowed the authors to review and classify them. Based on their analysis, three of the events attributed as AE may have been incorrectly attributed as a result of treatment (i.e. pre-existing constipation, common behavior of child, etc.). If these are excluded, the AE rate becomes one reaction per 1310 treatments.

The study by Miller and Benfield [16] is a higher quality study compared to the one by Hayes and Bezilla [30] because it incorporated a more rigorous methodological design. However, as a retrospective file review, there were likely to have been some methodological limitations such as reporting bias, different time periods over which the data was collected, and the difficulty of identifying adverse events experienced by infants but as reported by their parents.

Alcantara, Ohm and Kunz [14] conducted a retrospective cross-sectional survey of 577 pediatric patients (5,438 treatment visits) ranging from less than one day old to 18 years of age (mean age = 7.45; mode age = 1; median age = 7) attending for chiropractic treatment. All patients received treatment to at least one spinal region. Spinal manipulation was the most common treatment employed with 492 of the 577 pediatric patients received either manual (449) or instrumented mechanical manipulation (43).

In terms of adverse events, the parents of the pediatric patients reported only three events out of 5,438 treatments, all of which were minor (muscle stiffness, spine soreness, stiff and sore) and time limited. Based on these results, Alcantara et al [14] reported that 0.83% of pediatric patients or one in 1,812 patient visits resulted in a minor adverse event after chiropractic treatment. However this study was poorly designed with many possible sources of bias and errors. It suffers from similar methodological design flaws as the study by Hayes and Bezilla [30].

The three recent studies identified [14,16,30] were all retrospective file reviews and as such may have suffered from flaws associated with this methodology. Carnes et al. [12] suggest that several factors such as unclear definitions of Ped MT, different time periods of reporting, whether the patient or practitioner reports on the adverse events, confidentiality issues, missing data and missing data to follow-up, misinterpretation of data, quality assurance and bias of file reviewers, may adversely affect the validity of the study. Therefore the results of these three studies should be viewed with caution.

### A Recent Systematic Review of Adverse Events and Manual Therapy

Carnes et al [12] recently published a systematic review of adverse events and manual therapy (MT), irrespective of age. Inclusion criteria were studies which used manual therapy only; therapy was delivered by a registered therapist; the intervention was clearly described and adverse events were reported.

Carnes et al [12] identified eight prospective cohort studies and 31 manual therapy randomized controlled trials (RCTs). Although none of these studies specifically investigated Ped MT and adverse events, the results may provide a useful benchmark for pediatric adverse events in the absence of other research evidence. In addition, Carnes et al [12] performed a meta-analysis on the pooled data from both the cohort studies and the RCTs.

Carnes et al [12] were able to confirm that as reported by other authors, approximately half of adult patients treated by manual therapy are likely to experience a minor to moderate adverse event after treatment, and particularly after the first treatment [31-36]. These adverse events typically begin within 24 hours after treatment and most resolve within 48 hours [31,34]. However the risk of a SAE is small with no catastrophic adverse event such as death or stroke reported in any of the eight cohort studies or 31 RCTs included in the review.

In the meta-analysis, Carnes et al [12] were able to pool their results and compare MT to other therapies. MT was found to produce, in general, more adverse events compared to general medical practitioner care, about the same number compared to exercise but less adverse events than drug therapy (Non-steroidal anti-inflammatory drugs such as diclofenac or amitriptyline, an antidepressant).

Even though there were no reports of catastrophic or SAE in the 39 studies, one cannot assume that they did not occur as underreporting of adverse events is possible. Using the Exact method (according to binomial theory) Carnes et al [12] estimated the risk of an SAE after manual therapy at the upper 95% confidence interval to be approximately 0.13%. Thiel et al [33] using Handley's rule of three calculated an upper 95% confidence limit of SAE following chiropractic care in their RCT to be approximately 0.01%.

Carnes et al [12] concluded that the results of their meta-analysis showed that the relative risk of having a minor or moderate adverse event after high velocity thrust spinal manipulation was significantly less than taking medication that is often prescribed for these painful conditions.

### Limitations of the Systematic Review

The Carnes et al 2010 systematic review and meta-analysis [12] had some methodological limitations related to the methodological quality of the included studies. In particular, the included cohort and RCT studies may have suffered from unclear definitions of manual therapies, different time periods over which the data was collected, bias due to patient or practitioner reporting, confidentiality issues, patient satisfaction issues and loss of patients to follow-up. Patient reporting bias and patient selection bias may have also affected the findings, along with concurrent treatment with other healthcare professionals or self-medication by patients [12,33].

## Discussion

The application of manual therapy, particularly spinal manipulation to pediatric patients continues to be controversial. Of paramount importance to patients, parents, healthcare practitioners or the general public is the issue of safety and quality of care. The focus of this article was the issue of safety in terms of possible adverse events associated with pediatric manual therapy. A discussion of the appropriateness, efficacy or effectiveness is discussed elsewhere and is not in the scope of this report [7].

It is clear from a review of the literature that adverse events, including SAEs leading to permanent neurological disabilities, have been identified. Although SAEs involving death have also been reported, much of the data is sketchy at best with important information regarding type or schedule of treatment missing [see [15], Table one, page e278]. As tragic as an SAE involving death may be, it is of interest that such an event possibly associated with Ped MT has not been reported in the literature for almost 40 years [20]. Other SAEs which have resulted in permanent neurological consequences have been reported. However all of these were attributable to a misdiagnosis leading to the inappropriate application of SMT with unfortunate consequences. A narrative review by Pistolese in 1998 [37] of neurological or vertebrobasilar accidents after PSMT gave an estimate of one in 250 million PSMT. However this report has been criticized due to likely underreporting, although the authors do not offer any evidence for this opinion other than spontaneous reporting of AE likely leads to underreporting [15].

There are now three reports in the literature regarding prevalence or rates of adverse events associated with Ped MT as well as a recent systematic review of manual therapy [11,12,16,30].

Hayes and Bezilla [30] found that 9% of pediatric patients experienced an aggravation of their symptoms after Osteopathic SMT, none of which were serious complications,. Miller and Benfield [16] reported one in 100 patients, or a 1% rate, while Alcantara et al [14] reported a one in 1,812 patients or a 0.53% rate for AE and PSMT. It is possible that the higher AE rate in the Miller and Benfield [16] study could be attributed to pediatric patients being treated by chiropractic interns rather than fully qualified and experienced chiropractic practitioners and that AE were based on parents' report of crying which may be difficult to attribute solely as a result of PSMT [14]. Of primary importance is that none of the three studies reported SAEs and that all AEs identified were minor (mild signs or symptoms, transient, no specific medical intervention necessary). However, as discussed previously, retrospective file reviews are subject to a variety of biases and errors [12]. Consequently their results should be treated with caution. More, higher quality studies are needed to specifically address the issue of AE rates for pediatric manual therapy.

Nevertheless, it is interesting to note that AE rates for Ped MT are less than those for SMT in adult patients. From 30-56% of adult patients undergoing SMT report minor AE such as increased pain, stiffness, soreness, headache, etc.) compared to around 1% after Ped MT [32-36,38]. However, of note, no SAEs have been reported in any of the clinical studies for both adults and pediatric patients undergoing SMT [12,14,16,31,33-35]. More research is needed particularly in the pediatric population to confirm whether this is because MT is safe or because there is under reporting of adverse events [15,38,39].

The recent systematic review by Carnes et al [12] on adverse events and manual therapy, although focused on adverse events in all age groups after manual therapy, provides the most useful research information to date. The authors explored the incidence and risk of adverse events with manual therapies from a systematic review and meta-analysis of the current body of research literature on manual therapy.

The authors concluded that of the eight cohort studies and 31 RCTs included in their review, mild to moderate adverse events is common after MT; usually after the first treatment, occurring within 24 hours and resolving within 48 hours. However, serious adverse events are uncommon and when compared to other therapies for the same conditions, it was more than general practitioner care, the same as for exercise treatment but less than medication [12]. In particular, Based on best estimates, Carnes et al estimated that 0.13% of manual therapy treatments may result in a SAE [12]. The study by Thiel et al [33] estimated the risk of a SAE to occur in 0.01% of adult SMT treatments.

### Limitations of Current Evidence

All research is subject to limitations. Much more research is needed to better understand adverse events associated with pediatric manual therapy. In particular high quality studies directly focused on identifying adverse events with Ped MT are lacking. The best evidence currently comes from studies of adults treated by MT particularly SMT. There are no similar high quality studies of pediatric patients and MT.

As identified above, there are a number of possible methodological problems with investigating Ped MT in general as well as adverse events in particular. The categorization of manual therapy is broad and includes advice, touch, massage, mobilization as well as high velocity manipulation. This creates problems with identifying exactly what treatment or treatments have been given and how to compare them. Time between treatments also provides challenges in order to compare treatment outcomes as well as treatment schedules. Other problems include patients who are being co-treated by other healthcare practitioners and/or self-medicating. Patient or practitioner bias is also a major factor as the patient may feel compelled to under report adverse events and over report positive treatment outcomes. On the other hand, some patients whose treatment expectations have not been achieved may over report adverse events and under report any improvement.

Recording and reporting patient outcomes and adverse events also pose potential problems such as missing or incomplete data; inaccurate transcription or reporting of data and the use of outcome measures that are insufficient in terms of validity, reliability and clinical responsiveness.

It is clear that more high quality clinical studies are needed specifically designed to investigate adverse events in pediatric manual therapy. Until better research is available, patients and clinicians must rely mainly on the evidence provided by studies of adverse events associated with manual therapy in adults.

## Conclusions

The purpose of this report was to review the current state of evidence regarding possible adverse events associated with pediatric manual therapy. Unfortunately very few high quality studies are currently available in this area. Most evidence comes from studies on adult patients and spinal manipulative therapy. From these studies, current evidence suggests that minor or moderate adverse events after manual therapy are common but that serious adverse events are rare. Manual therapy such as spinal manipulation in adults appears to have significantly fewer serious side effects compared to medication but equal to exercise prescription. Much more research specifically directed at identifying possible adverse events associated with pediatric manual therapy is needed.

## Competing interests

The author declares that he has no competing interests.
